# Defining antimicrobial susceptibility testing methods and breakpoints among *Achromobacter* species

**DOI:** 10.1128/jcm.00264-25

**Published:** 2025-04-30

**Authors:** Harley Harris, Haley Stambaugh, Emily Jacobs, Amira Bhalodi, Sukantha Chandrasekaran, Nicolynn C. Cole, Jennifer Lu, Tsigereda Tekle, Romney Humphries, Patricia J. Simner

**Affiliations:** 1 Department of Pathology, Division of Medical Microbiology, Johns Hopkins University School of Medicine 1500, Baltimore, Maryland, USA; 2 Scientific and Medical Affairs Consulting LLC, Newton, Pennsylvania, USA; 3 Department of Pathology and Laboratory Medicine, University of California, Los Angeles 166650, Los Angeles, California, USA; 4 Department of Laboratory Medicine and Pathology, Mayo Clinic 6915, Rochester, Minnesota, USA; 5 Department of Pathology, Microbiology, and Immunology, Division of Laboratory Medicine, Vanderbilt University Medical Center 12328, Nashville, Tennessee, USA; Children's Hospital Los Angeles, Los Angeles, California, USA

**Keywords:** *Achromobacter*, breakpoints, antimicrobial susceptibility testing, disk diffusion, minimal inhibitory concentrations, epidemiological cutoff values

## Abstract

*Achromobacter* species can cause opportunistic infections which are difficult to treat due to a variety of antimicrobial resistance (AMR) mechanisms. As such, antimicrobial susceptibility testing (AST) is a cornerstone to successful treatment for these organisms. Currently, there are no specific Clinical and Laboratory Standards Institute (CLSI) AST guidelines for *Achromobacter* species. The purpose of this study was to (i) establish tentative CLSI M45 MIC and disk diffusion (DD) breakpoints (BPs), (ii) evaluate the modified carbapenem inactivation method (mCIM), and (iii) understand the mechanisms mediating AMR among *Achromobacter* species. Contemporary MIC data from multiple sources were collated to define the tentative epidemiological cutoff values (tECVs), and the MIC distributions were used to inform the establishment of MIC BPs for various agents. A disk-to-MIC correlate study with 91 isolates from across the United States was completed by testing reference broth microdilution and DD from the same inoculum and applying the dBETS software to establish DD BPs. Lastly, the mCIM and whole-genome sequencing (WGS) were pursued. The tECVs for piperacillin-tazobactam, imipenem, and meropenem were 1, 2, and 0.5 µg/mL, respectively. Disk correlates met CLSI M23 acceptance criteria with a few exceptions related to a small number of isolates, resulting in high minor errors. WGS revealed that 82 (90.1%) isolates harbored a *bla*_OXA_ variant with *bla*_OXA-114_ predominating (90.2%). Nineteen isolates harbored acquired beta-lactamase genes, including 16 *bla*_AXC_, 2 *bla*_VIM-4_, and 1 *bla*_AZM-1._ The mCIM had a sensitivity of 100% and specificity of 87%. Upon review, the CLSI M45 committee set tentative MIC and DD BPs for piperacillin-tazobactam, imipenem, meropenem, and trimethoprim-sulfamethoxazole.

**IMPORTANCE:**

*Achromobacter* species can cause opportunistic infections which may be difficult to treat due to a variety of antimicrobial resistance mechanisms. Antimicrobial susceptibility testing is a critical component of patient treatment for these infections. Currently, the Clinical and Laboratory Standards Institute M100 non-Enterobacterales susceptibility test interpretive criteria and methodology are utilized for *Achromobacter* by clinical laboratories and likely do not accurately predict susceptibility results for *Achromobacter* species. This study was designed to establish tentative MIC and disk diffusion breakpoints specific to *Achromobacter* species.

## INTRODUCTION

*Achromobacter* species belong to the family *Alcaligenaceae* within the order *Burkholderiales* (1). Currently, the genus *Achromobacter* has 22 members (2). *A. xylosoxidans* is the most common species recovered from clinical samples worldwide (3). Distribution of other species varies geographically, with *A. dolens* and *A. insuavis* being more prevalent in Europe and *A. ruhlandii* being the second most common in the Americas (3).

*Achromobacter* species are environmental, glucose non-fermenting, gram-negative bacilli that can cause a broad range of opportunistic infections, in particular respiratory tract infections. Risk factors for infection include cystic fibrosis, the presence of indwelling devices (e.g., catheters and endotracheal tubes), other underlying medical comorbidities (e.g., diabetes mellitus, chronic renal failure, and chronic heart disease), and current or previous hospitalization or health care exposure (3). A common feature of *Achromobacter* species is the broad range of antimicrobial resistance (AMR) through intrinsic and acquired mechanisms, which contribute to the difficulty in effectively managing infections caused by these pathogens. As such, antimicrobial susceptibility testing (AST) is a cornerstone to successful treatment of infections caused by these multidrug-resistant organisms.

The current Clinical and Laboratory Standards Institute (CLSI) AST guidelines for *Achromobacter* species apply the M100 non-Enterobacterales breakpoints (BPs) that are used for all non-fastidious, non-glucose fermenting gram-negative bacilli without specific BPs. This approach is associated with several limitations. CLSI non-Enterobacterales BPs have not been updated since their initial publication, despite several revisions to the predicate CLSI *Pseudomonas aeruginosa* and Enterobacterales BPs, based on emerging pharmacokinetic-pharmacodynamic (PK/PD) data and novel AMR mechanisms (e.g., carbapenemases). As a result, the non-Enterobacterales BPs likely provide suboptimal estimates of susceptibility for the non-Enterobacterales and certainly require review for potential revision. Furthermore, only minimal inhibitory concentration (MIC) BPs are available for the non-Enterobacterales (4), precluding the use of the standard disk diffusion (DD) method. DD is commonly employed by laboratories in the absence of an FDA-cleared, automated MIC method. Furthermore, the performance characteristics of commercial methods for *Achromobacter* are unknown but may be poor, as was recently shown for *Stenotrophomonas maltophilia* and *Burkholderia cepacia* complex, two glucose-non-fermenting organisms where laboratories utilize commercial AST systems off-label (5–8). Additionally, the non-Enterobacterales organism group encompasses several genera, resulting in BPs for antimicrobials to which *Achromobacter* are intrinsically resistant (e.g., aminoglycosides, cephalosporins [except ceftazidime], and aztreonam) (3, 9). These antimicrobials may be reported as susceptible in error due to ambiguity in the BPs, compounded by uncertainty regarding AST accuracy as described above. Lastly, the CLSI M100 non-Enterobacterales BPs are not recognized by the US Food and Drug Administration, and therefore, no new FDA-cleared diagnostics are possible, as the application of FDA BPs has been a requirement for the clearance of an AST device since 2006 (10). Unlike CLSI, the European Committee on Antimicrobial Susceptibility Testing (EUCAST) has set *A. xylosoxidans* specific BPs for piperacillin-tazobactam, meropenem, and trimethoprim-sulfamethoxazole.

This study was conducted to review antimicrobial agent MIC data sets for contemporary isolates, generate *in vitro* reference broth microdilution (BMD) MIC distributions, and DD correlates to support the creation of tentative CLSI M45 BPs for *Achromobacter* species. Furthermore, whole-genome sequencing (WGS) data were pursued to evaluate the genomic correlates of AMR among a contemporary set of *Achromobacter* isolates collected from across the United States and to evaluate the modified carbapenem inactivation method (mCIM) for carbapenemase detection.

## MATERIALS AND METHODS

### Establishing M45 clinical BPs

The process to establish BPs for the CLSI M45 guideline is different from the M100 standard, which follows M23 guidance (4, 11, 12). For M45 organisms, the data to establish clinical BPs are not as robust; in particular, PK/PD and clinical outcome data are rarely available. Thus, a less stringent approach is followed to evaluate setting BPs for M45 organisms, including *Achromobacter* species. Contemporary MIC data were collated from publications, large databases, and reference laboratories. Histograms were created with the MIC data to compare with the current non-Enterobacterales M100 BPs, BPs from related organisms (*Pseudomonas aeruginosa, Acinetobacter* species, or Enterobacterales) or BPs from other standards development organizations (e.g., EUCAST, Table 1).

**TABLE 1:** Clinical breakpoints and/or epidemiologic cutoff values for gram-negative organisms evaluated to develop tentative clinical breakpoints for *Achromobacter* species^*a*^

	CLSI M100-S34 Enterobacterales	CLSI M100-S34 non-Enterobacterales	CLSI M100-S34 *Pseudomonas aeruginosa*	CLSI M100-S34 *Acinetobacter* species	EUCAST 2024 v.14 *Achromobacter xylosoxidans*
Piperacillin-tazobactam	S: ≤8/4 SDD: 16/4 R: ≥32/4	S: ≤16/4 I: 32/4–64/4 R: ≥128/4	S: ≤16/4 I: 32/4 R: ≥64/4	S: ≤16/4 I: 32/4–64/4 R: ≥128/4	ECV: 4 S: ≤4 R: ≥8
Imipenem	S: ≤1 I: 2 R: ≥4	S: ≤4 I: 8 R: ≥16	S: ≤2 I: 4 R: ≥8	S: ≤2 I: 4 R: ≥8	Not established
Meropenem	S: ≤1 I: 2 R: ≥4	S: ≤4 I: 8 R: ≥16	S: ≤2 I: 4 R: ≥8	S: ≤2 I: 4 R: ≥8	ECV: 1 S: ≤1 I: 2 R: ≥4
Trimethoprim-sulfamethoxazole	S: 2/38 R: 4/76	S: 2/38 R: 4/76	S: 2/38 R: 4/76	S: 2/38 R: 4/76	WT MICs: ≤0.06 S: ≤0.125 R: ≥0.25
Ceftazidime	S: ≤4 I: 8 R: ≥16	S: ≤8 I: 16 R: ≥32	S: ≤8 I: 16 R: ≥32	S: ≤8 I: 16 R: ≥32	Not established
Ciprofloxacin	S: ≤0.25 I: 0.5 R: ≥1	S: ≤1 I: 2 R: ≥4	S: ≤0.5 I: 1 R: ≥2	S: ≤1 I: 2 R: ≥4	Not established
Levofloxacin	S: ≤0.5 I: 1 R: ≥2	S: ≤2 I: 4 R: ≥8	S: ≤1 I: 2 R: ≥4	S: ≤2 I: 4 R: ≥8	Not established
Minocycline	S: ≤4 I: 8 R: ≥16	S: ≤4 I: 8 R: ≥16	IR	S: ≤4 I: 8 R: ≥16	Not established

^*a*^ S, susceptible; SDD, susceptible dose-dependent; I, intermediate; R, resistant; IR, intrinsic resistance; ECV, epidemiological cutoff value; WT, wild type.

### MIC distributions and epidemiological cutoff values

*Achromobacter* contemporary MIC data sets were obtained from three sources: (i) AST data performed on isolates collected between 2015 and 2021 at University of California, Los Angeles (UCLA), generated by reference BMD (*N*: 711); (ii) AST data performed on isolates collected between 2015 and 2021 from the Mayo Clinic in Rochester, MN generated by agar dilution (*N*: 1,639); and (iii) AST data performed on isolates collected between 2013 and 2022 from the SENTRY database generated by reference BMD, available at sentry-mvp.jmilabs.com (*N*: 548). Histograms were plotted by the number of isolates at each MIC for each individual data set and as a combined data set. Of note, differences in the range of doubling dilutions tested for each antimicrobial agent were apparent between testing sites based on their standard operating procedures. The tentative epidemiological cutoff values (tECVs) for each antimicrobial agent were determined to estimate the upper end of the wild-type distribution using the ECOFF finder with a 97.5% probability threshold (13).

### Establishing MIC clinical BPs

As there is little PK/PD and clinical outcomes data available for *Achromobacter*, PK/PD was extrapolated from reasonable surrogate species (e.g., *P. aeruginosa* for the beta-lactams and Enterobacterales for trimethoprim-sulfamethoxazole) and evaluated relative to the MIC distributions. Extrapolation of PK/PD is imperfect, and as such, the tECV was used to help set the clinical BP, provided it was predicted to achieve target attainment in PK/PD assessment for the surrogate organisms (Table 1).

### Antimicrobial susceptibility and antimicrobial resistance testing

To establish disk correlates and evaluate mechanisms of AMR, 91 contemporary, geographically diverse isolates of *Achromobacter* species were collected from three US clinical laboratories (Johns Hopkins University [JHU], UCLA, and ARUP Laboratories) and the US Centers for Diseases Control and Prevention (12). Isolates were randomly selected from the sites.

To establish DD BPs for AST of *Achromobacter* species, reference BMD and DD methods were performed in parallel at a single testing site. Both AST methods were performed from the same bacterial inoculum standardized to a suspension equivalent to a 0.5 McFarland standard. Reference BMD was performed in duplicate using the same inoculum following CLSI M07 guidelines, incubated in ambient air at 35°C ± 2°C and read after 16–20 h of incubation (14). The average of the two BMD results, which was arbitrarily rounded down to the nearest twofold dilution, was used as the reference MIC. The following agents were included on the panel: ceftazidime (2–32 µg/mL), ciprofloxacin (0.125–8 µg/mL), imipenem (0.25–16 µg/mL), levofloxacin (0.25–16 µg/mL), meropenem (0.25–16 µg/mL), minocycline (1–16 µg/mL), piperacillin-tazobactam (4–128 µg/mL), and trimethoprim-sulfamethoxazole (0.5–8 µg/mL). Mueller Hinton Broth (Difco, BD) was cation-adjusted, and antimicrobial powders were obtained from US Pharmacopeia (USP). DD testing (BD BBL Sensi-Disc, Sparks, MD) was performed following CLSI M02 guidelines and incubated in ambient air at 35°C ± 2°C and read after 16–18 h of incubation on MHA from Remel (15). The zone diameters were recorded at 100% inhibition, with the exception of trimethoprim-sulfamethoxazole, which was read at 80% inhibition. Daily QC was set up for both BMD and DD testing using *Pseudomonas aeruginosa* ATCC 27853 and *Klebsiella pneumoniae* ATCC 700603.

Using the AST data generated from the 91 *Achromobacter* isolates, a scattergram was created for each antimicrobial to visualize the DD to MIC correlates. Using the tentative MIC BPs as described above, the error-bounded method was used to select the zone diameter BPs and to calculate discrepancy rates between MIC and zone diameter test results as described by CLSI. The dBETS tool was applied to generate DD BPs (http://dbets.shinyapps.io/dBETS/). Categorical agreement (CA), major errors (MEs), very major errors (VMEs), and minor errors (MIs) were evaluated as appropriate. CA is defined as the agreement of interpretative results between DD and BMD using the newly defined BPs (as previously described). Discrepancies between DD and BMD were categorized as follows: VME, susceptible result by DD and resistant by BMD; ME, resistant result by DD and susceptible by BMD; MI, a discrepancy between DD and BMD involving an intermediate (I)/susceptible dose-dependent (SDD) category. Any ME and VME results were repeated to see if errors would resolve. Acceptable VME, ME, and MI rates for isolates in the I *±* 1 category (i.e., isolates with BMD MIC values within one doubling dilution of the intermediate/SDD BP) were <10%, <10%, and <40%, respectively . For isolates in the ≤I − 2 or ≥I + 2 categories with BMD MIC values outside one doubling dilution of the I/SDD BP, acceptable VME, ME, and MI rates were <2%, <2%, and <5%, respectively (12).

### Carbapenemase detection

To evaluate the mCIM for detection of carbapenemase production among *Achromobacter* species, the mCIM was performed using a 10 µL inoculum from the purity plate following the AST study (4, 16, 17).

### WGS

All isolates were sequenced at JHU. Using the same bacterial inoculum that was used for the AST study, genomic DNA (gDNA) was extracted from pure cultures using the DNeasy Power Biofilm Kit (Qiagen). Approximately 100–500 ng of gDNA was used to prepare sequencing libraries using the Illumina DNA Prep Kit. Sequencing was performed on an Illumina NextSeq platform, to a target goal of 50× sequencing depth by Illumina. The Illumina paired reads were processed using the PHoeNIx pipeline (18) for read filtering, assembly, and classification. The pipeline automates BBDuk (19) for the removal of PhiX174 reads/adaptors, fastp (20) for quality filtering and trimming, SPAdes (21) for genome assembly, SRST2 (22) for MLST, Kraken2 (23) for classification, and finally GAMMA (24) for AMR gene identification.

## RESULTS

### MIC breakpoint determination

MIC data were analyzed separately and in aggregate to visualize the MIC distributions and to attempt to establish a tECV. The epidemiological cutoff values (ECVs) were considered tentative as not all M23 criteria were met to establish true ECVs (e.g., use of a recognized reference method, data from ≥3 laboratories, on-scale MICs). The MIC distributions and tECVs for antimicrobial agents for which tentative clinical BPs were established are summarized in Table 2 and Supplementary Material 1. For meropenem and imipenem, the tECV was found to be 0.5 µg/mL and 2 µg/mL, respectively. The clinical BPs were adapted from those of CLSI M100-S34 *P. aeruginosa* and *Acinetobacter* species, and the proposed BPs were set at susceptible ≤2 µg/mL, intermediate 4 µg/mL, and resistant ≥8 µg/mL for both carbapenems. For piperacillin-tazobactam, the tECV was 1 µg/mL. Based on PK/PD data evaluated by CLSI for the Enterobacterales and *P. aeruginosa* BP revisions in 2019 and 2020, it was decided to utilize a susceptible/SDD/resistant BP adapted from the Enterobacterales BPs and based on the same dosing strategies (25). Thus, the BPs for piperacillin-tazobactam were set as ≤8 (susceptible)/16 (SDD)/≥32 µg/mL (resistant). The susceptible breakpoint was established based on a dosing regimen of 3.375–4.5 g administered every 6 h as a 30 min infusion, and the SDD breakpoint was established based on a dosing regimen of 4.5 g administered every 6 h as a 3 h infusion, or 4.5 g administered every 8 h as a 4 h infusion. Trimethoprim-sulfamethoxazole was more challenging to set a tECV as dilution ranges for the contemporary data sets did not include MICs below 0.5 µg/mL, and most of the population had MICs ≤0.5 µg/mL. EUCAST reported in a small study that the wild-type population demonstrated an MIC ≤0.06 µg/mL (26). Based on the EUCAST data and contemporary distribution data, the tentative BP was proposed to be at ≤2 µg/mL susceptible and ≥4 µg/mL resistant, which is consistent with other CLSI M100 gram-negative organisms with trimethoprim-sulfamethoxazole BPs (e.g., the Enterobacterales, *Acinetobacter* spp., other non-Enterobacterales, and *S. maltophilia*).

**TABLE 2:** Contemporary *Achromobacter* species MIC data from three sites for various antimicrobial agents^*a*^^,^^*c*^

Antimicrobial agent	Dilution (μg/mL)														Total
	0.03	0.06	0.12	0.25	0.5	1	2	4	8	16	32	64	128	>	
P-T AD										1,478	45	34		84	1,641
P-T BMD 1									640	23	6	4	4	33	710
P-T BMD 2					271	132^*b*^	31	20	21	20	20	10		23	548
P-T ALL										2,636	71	48		144	2,899
IMI BMD 1				2	27	322	209^*b*^	74	25	12				24	695
IMI BMD 2			1	6	94	261	49^*b*^	72	28					37	548
IMI-ALL				9	121	583	258^*b*^	146	53					73	1,243
MER AD			210	NT	NT	675	126	102	82					143	1,338
MER BMD 1				340	69^*b*^	51	62	91	43	25				30	711
MER BMD 2	5	35	181	71^*b*^	48	39	32	54	19	21	9			34	548
MERO-All				842	117^*b*^	765	220	247	144					262	2,597
SXT AD 1					1,351	0	106							179	1,636
SXT BMD 1					4	640	10	10						48	712
SXT BMD 2					421	31	23	19						54	548
SXT-ALL					1,776	671	139							310	2,896
CAZ AD 1								696	407	233				300	1,636
CAZ BMD 1						22	56	202	173	134	57			66	710
CAZ BMD 2			1	1	1	11	94	194	115	66	27			38	548
CAZ-ALL								1,278	695	433				488	2,894
CIP AD 1				1	3	90	234							1,308	1,636
CIP BMD 1				3	3	23	3							481	513
CIP BMD 2	2	2	0	0	0	26	87	172						259	548
CIP-ALL				8	6	139	324							2,220	2,697
LEV AD 1					9	151	275	347						560	1,342
LEV BMD 1							248	131	133					196	722
LEV BMD 2			3	0	7	57	137	149						195	548
Levofloxacin-ALL							887	627						1,084	2,612
MIN BMD 1					3	1	10	12	5					3	34
MIN BMD 2		3	9	31	79	162	125	72	23					7	511
MIN-ALL						163	135	84	28					10	545

^*a*^ NT, dilution not tested; AD, agar dilution; BMD, broth microdilution; BMD 1, testing site 1; BMD 2, testing site 2; P-T, piperacillin-tazobactam; IMI, imipenem; MER, meropenem; SXT, trimethoprim-sulfamethoxazole; CAZ, ceftazidime; CIP, ciprofloxacin; LEV, levofloxacin; MIN, minocycline.

^*b*^ tECV, tentative epidemiological cutoff value.

^*c*^ Empty cells indicate that the particular dilution was not tested.

MIC distributions for ceftazidime, ciprofloxacin, levofloxacin, and minocycline were also evaluated (Table 2). Modal MICs were above the MICs typically predicted to be achievable for similar organisms when using routine antimicrobial dosing, indicating limited clinical activity for these antimicrobial agents (Table 2). Thus, BPs for these agents were not set by the working group.

### Disk diffusion correlates

BMD and DD were performed on a collection of 91 isolates to establish DD BPs (Table 3). DD correlates to MIC were assessed using the newly determined BPs (Table 4), by testing isolates by DD and BMD in parallel, using the same inoculum. Best-fit DD correlates are summarized in Table 5. All results were within M23 acceptance criteria, using the error-rate bounded method with a few exceptions involving minor errors. For example, 45.5% and 50% minor errors occurred in the I + 1 and I − 1 range for meropenem and piperacillin-tazobactam, respectively. For trimethoprim-sulfamethoxazole, ≥I + 2 and ≤I − 2 were both above the acceptable threshold of <5%, with minor error rates of 20% for ≥I + 2 and 7.6% for ≤I − 2. The committee found these exceptions acceptable due to the small denominator of isolates and only involving minor errors.

**TABLE 3:** *Achromobacter* species, multi-locus sequence types, and antimicrobial resistance genes among isolates included in the disk correlate study

*Achromobacter* species	Number of isolates (%)	MLST (number of isolates)	Beta-lactamase genes (*n*, %)
*Achromobacter* *xylosoxidans*	77 (84.6%)	Novel profile/allele (41), ST175 (8), ST509 (3), ST184 (2), ST176 (2), 1 of each ST19, ST20, ST21, ST236, ST27, ST73, ST28, ST303, ST426, ST433, ST438, ST448, ST73, ST173, ST180, ST182	*bla*_OXA-114_ variants (53), *bla*_AXC-1_ & *bla*_OXA-114_ (10), *bla*_AXC-3_ & *bla*_OXA-258_ (4), *bla*_OXA-243_ (3), *bla*_OXA-114_ *& bla*_VIM-4_ (2), *bla*_AXC-5_ & *bla*_OXA-114_ (1), *bla*_AXC-7_ & *bla*_OXA-114_ (1), none (3)
*Achromobacter deleyi*	8 (8.8%)	Novel profile/allele (5), ST461 (1), ST54 (1), ST55 (1)	*bla*_OXA-364_ (7), *bla*_OXA-789_ (1)
*Achromobacter* species, not further speciated	2 (2.2%)	Novel profile/allele (2)	None
*Achromobacter spanius*	1 (1.1%)	Novel profile/allele	None
*Achromobacter mucicolens*	1 (1.1%)	Novel profile/allele	*bla*_AMZ-1_
*Achromobacter insolitus*	1 (1.1%)	Novel profile/allele	None
*Achromobacter* *denitrificans*	1 (1.1%)	Novel profile/allele	None
Total	91		

**TABLE 4:** Clinical and Laboratory Standards Institute M45 4th edition tentative epidemiological cutoff values and clinical breakpoints for *Achromobacter* species

	ECV (µg/mL)	MIC (µg/mL)					Disk diffusion (mm)		
		S	I	SDD	R	S	I	SDD	R
Piperacillin-tazobactam	1	≤8/4		16/4	≥32/4	≥28		23–27	≤22
Imipenem	2	≤2	4		≥8	≥24	18–23		≤17
Meropenem	0.5	≤2	4		≥8	≥21	16–20		≤15
Trimethoprim-sulfamethoxazole		≤2			≥4	≥28	21–27		≤20

**TABLE 5:** Disk diffusion correlates to reference BMD MIC results

Antimicrobial (disk content) S/I/R disk breakpoint in mm	Range	N	CA	VME	ME	MI
Piperacillin-tazobactam (100/10 µg) ≥28/23–27/≤22	≥I + 2	4	4	0		0
	I + 1 & I − 1	2	1	0	0	1 (50%)
	≤I − 2	84	83		0	1 (1.2%)
	Total	90	88 (97.8%)	0	0	2 (2.2%)
Imipenem (10 µg) ≥24/18–23/≤17	≥I + 2	7	7	0		0
	I + 1 & I − 1	36	34	0	0	2 (5.6%)
	≤I − 2	47	47		0	0
	Total	90	88 (97.8%)	0	0	2 (2.2%)
Meropenem (10 µg) ≥21/16–20/≤15	≥I + 2	7	7	0		0
	I + 1 & I − 1	11	6	0	0	5 (45.4%)
	≤I − 2	72	72		0	0
	Total	90	85 (94.4%)	0	0	5 (5.6%)
Trimethoprim-sulfamethoxazole (1.25/23.75 µg) ≥28/21–27/≤20	≥I + 2	5	4	0		1 (20%)
	I + 1 & I − 1	5	4	0	0	1 (20%)
	≤I − 2	79	72		1 (1.26%)	6 (7.6%)
	Total	89	80 (89.9%)	0	1 (1.25%)	8 (9.0%)

### Whole-genome sequencing results

WGS was performed to confirm the species identification of the isolates and to determine genetic correlates of resistance. The identity of the *Achromobacter* species, multilocus sequence type (MLST) and beta-lactamase genes detected by WGS is summarized in Table 3. The most common species was *A. xylosoxidans* (77; 84.6%) followed by *A. deleyi* (8; 8.8%) with a handful of other species encountered (*A. mucicolens, A. spanius, A. insolitus*, and *A. denitrificans*).

Of our tested isolates, 82 (90.1%) harbored a *bla*_OXA_ variant, with most *A. xylosoxidans* (74; 96.1%) harboring a variant of *bla_OXA-114_* gene (67; 87%), *bla*_OXA-258_ (4; 5.4%), or *bla*_OXA-243_ (3; 4.1%). Eighteen *A*. *xylosoxidans* also harbored another beta-lactamase gene, including 16 *bla*_AXC_ and 2 *bla*_VIM-4_. All eight *A*. *deleyi* isolates harbored a *bla*_OXA-243-like_ variant, including *bla*_OXA-364_ (7) and *bla*_OXA-789_ (1). The other *Achromobacter* species did not harbor a detected beta-lactamase gene with the exception of *A. mucicolens* that harbored *bla*_AMZ-1_.

### Modified carbapenem inactivation method results

Of the isolates evaluated, 13 (14.3%) were found to be not susceptible to either meropenem or imipenem. Based on the WGS data, the sensitivity and specificity of the mCIM were 100% and 87%, respectively, for the presence of carbapenemase. The mCIM was positive for eight *A*. *xylosoxidans* isolates, including the two true positive VIM producers and six false positive AXC producers. All other isolates were mCIM negative, including the remaining 10 AXC producers and the one AMZ producer.

## DISCUSSION

*Achromobacter* species can cause opportunistic infections with limited treatment options due to a multitude of intrinsic and acquired AMR mechanisms. As such, AST is critical to guide treatment decisions for infections with these multidrug-resistant organisms. In this study, CLSI M45 tentative MIC and DD BPs for *Achromobacter* spp. were established for four antimicrobials: meropenem, imipenem, piperacillin-tazobactam, and trimethoprim-sulfamethoxazole (Table 4). The agents align with those that are proposed by EUCAST with the exception of imipenem (27). The breakpoints proposed herein are considered tentative until officially published in the 4th edition of the M45 document. For imipenem, EUCAST reviewed data for 92 isolates but found that the tECV (8 µg/mL) was too high to set BPs (26, 28). In contrast, MIC distributions of over 500 isolates from the North American SENTRY database were evaluated, and the tECV was 2 µg/mL. Thus, the M45 committee decided to pursue a tentative imipenem BP as it is achievable with routine dosing regimens and supported by surrogate organism BPs. Differences in meropenem and imipenem tECVs are not well understood but may be related to differences in substrate specificities of efflux pumps and/or porins harbored by *Achromobacter* species (3).

Furthermore, in January 2025, EUCAST added cefiderocol MIC and DD testing guidance for *Achromobacter xylosoxidans*, applying similar methods developed for other aerobic gram-negative bacilli (e.g., iron-depleted cation-adjusted Mueller Hinton broth). No BPs are provided. However, they indicate that an MIC of ≤0.5 mg/L (zone diameter ≥ 26 mm) is mostly devoid of AMR mechanisms. The CLSI M45 committee did not evaluate cefiderocol in this study.

Despite being used clinically, ceftazidime, the fluoroquinolones (ciprofloxacin and levofloxacin), and minocycline BPs were not able to be defined in this study. The antimicrobial agent MICs for most wild-type isolates were above the MICs typically achievable by routine antimicrobial dosing for similar organisms, indicating limited activity of the antimicrobial agents (29). These findings are similar to those reported by EUCAST and the reason why both standards development organizations did not pursue BPs for these agents. Further complicating treatment is the recognition that *Achromobacter* is intrinsically resistant to ampicillin, amoxicillin, cefotaxime, ceftriaxone, aztreonam, ertapenem, the aminoglycosides, and colistin (3, 9). New intrinsic resistance tables will be introduced in the forthcoming CLSI M45 4th edition to help users identify agents where organisms are intrinsically resistant or for which wild-type MICs may be higher than typically achievable by routine dosing.

Although the great majority of the data reviewed by the CLSI M45 committee were for *A. xylosoxidans*, identification of *Achromobacter* to the species level can be difficult by phenotypic methods and is dependent on MALDI-TOF MS databases (30, 31). This is further supported by this study, where many isolates in the contemporary data set and the disk correlate study were submitted with a genus-level ID only. WGS was able to confirm that the majority of isolates submitted for the disk correlate study as *A. xylosoxidans* with *A. deleyi* being the second most common species. This is in contrast to other published reports that state that *A. ruhlandii* is the second most common in the Americas. The data from non-*xylosoxidans Achromobacter* species evaluated suggest that the tentative BPs can be applied to other species. As such, the committee decided to develop the BPs for the genus *Achromobacter* and not further specify to the species level, as was pursued by EUCAST.

WGS revealed that 90.1% of isolates harbored a *bla*_OXA_ variant with *bla*_OXA-114_-like predominating (90.2%). In this data set, a total of 14% of isolates were carbapenem not susceptible based on the newly defined BPs. Only 2 (15%) of the carbapenem not susceptible isolates harbored a known metallo-beta-lactamase, *bla*_VIM-4_. Additional beta-lactamase genes identified included *bla*_AXC_ variants and *bla*_AMZ_. Although *bla*_AXC_ genes have been associated with carbapenem resistance, kinetic studies have not been pursued to confirm that carbapenems are a suitable substrate for the beta-lactamase genes. Furthermore, the presence of the gene does not always correlate with carbapenem resistance (32, 33). Thus, it appears that the majority of carbapenem resistance is mediated by non-carbapenemase mechanisms and is likely due to multidrug-resistant efflux pumps, as previously reported (3). It is important to note that the newer beta-lactam-beta-lactamase inhibitor combinations, such as ceftolozane-tazobactam, ceftazidime-avibactam, and meropenem-vaborbactam, will not overcome these carbapenem resistance mechanisms. Overall, the mCIM had a sensitivity of 100% and specificity of 87%. Due to the limited number of carbapenemase producers in the study, further evaluations of the mCIM are required to more robustly evaluate its performance for use with *Achromobacter*. Based on the preliminary results generated here, the data suggest that the mCIM may be used as a screen for carbapenemase production, but if positive, the result would need to be confirmed by a secondary method (e.g., molecular method) due to the specificity issues.

There are some limitations to this study. First, the BPs developed are based almost entirely on MIC distribution data. Typically, PK/PD studies, patient outcomes, and large MIC data sets are used to establish AST BP standards (12). Because this depth of data is not available for *Achromobacter* spp., we are recommending these BPs be tentative guidelines for testing and will be submitted for inclusion in the 4th edition of the CLSI M45 guideline. Another limitation is that the DD correlate study was conducted at a single center using a single lot and manufacturer of disks and media. While appropriate quality controls were used to ensure test accuracy, the impact of site-to-site variability or manufacturer differences is not known.

In summary, we present the data used by the volunteers tasked by CLSI to develop *Achromobacter* species BPs in the M45 guideline. Upon review, the CLSI M45 committee proposed tentative MIC and DD BPs for piperacillin-tazobactam, imipenem, meropenem, and trimethoprim-sulfamethoxazole. Laboratories may review the data presented here with their antimicrobial stewardship committee to adopt the proposed tentative clinical breakpoints, or alternatively they can decide to wait until the official publication in the forthcoming 4th edition of the CLSI M45 document in early 2026.

## Data Availability

The WGS data were deposited to SRA under bioproject PRJNA1214843.

## SUPPLEMENTAL MATERIAL

Supplemental material jcm.00264-25-s0001.pdf MIC distributions figures.

ASM does not own the copyrights to Supplemental Material that may be linked to, or accessed through, an article. The authors have granted ASM a non-exclusive, world-wide license to publish the Supplemental Material files. Please contact the corresponding author directly for reuse.
