# Supplementary material for: Defining antimicrobial susceptibility testing methods and breakpoints among Achromobacter species
Source: J Clin Microbiol. 2025 Apr 30;63(6):e00264-25. doi: 10.1128/jcm.00264-25 (PMC12153331; doi:10.1128/jcm.00264-25)
Supplement: Supplemental material — MIC distributions figures. [file jcm.00264-25-s0001.pdf]

Achromobacter spp. Imipenem MIC data (n = 1243)

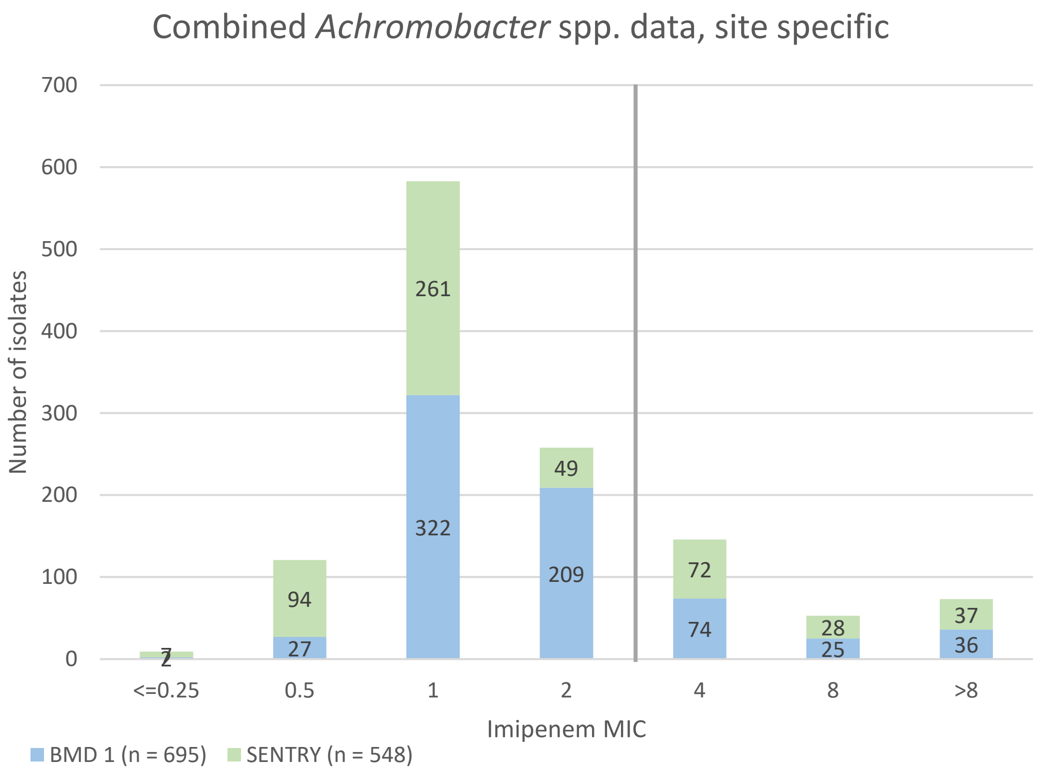

ECOFF Finder @ 97.5%: 2 ug/ml

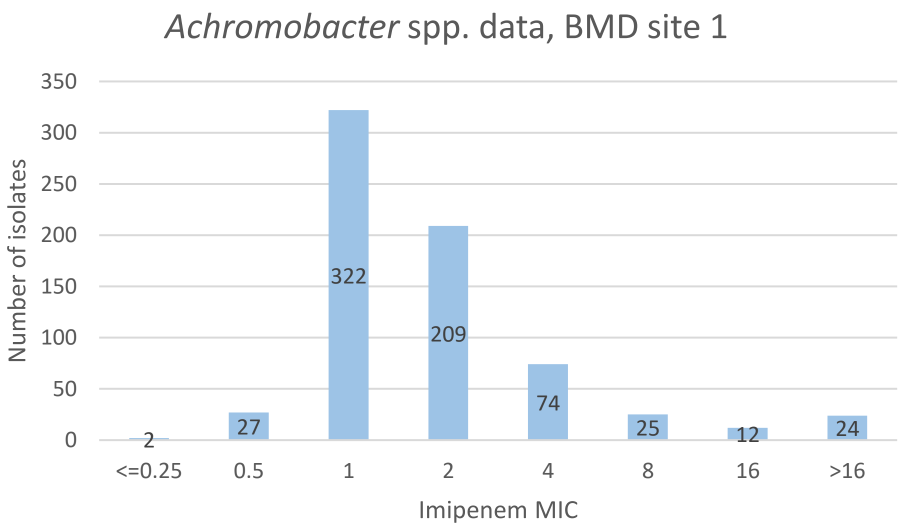

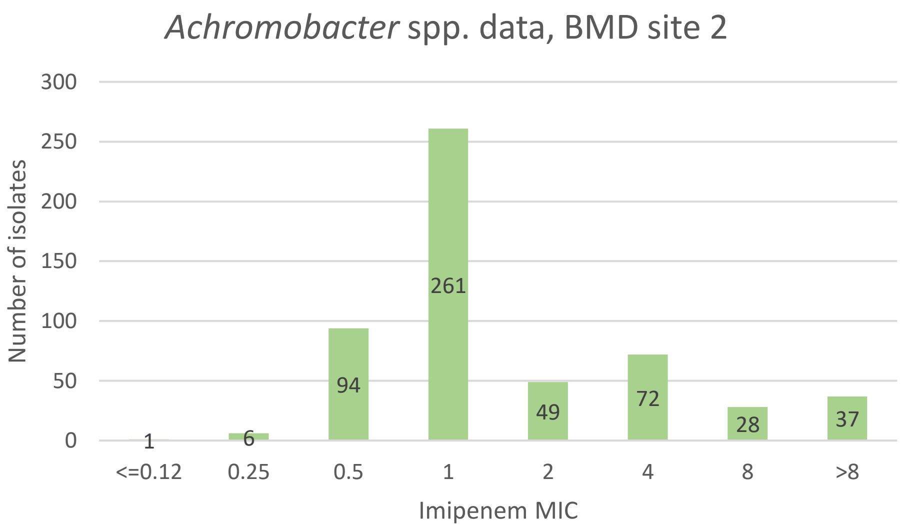

Achromobacter spp. Meropenem MIC data (n = 2597)

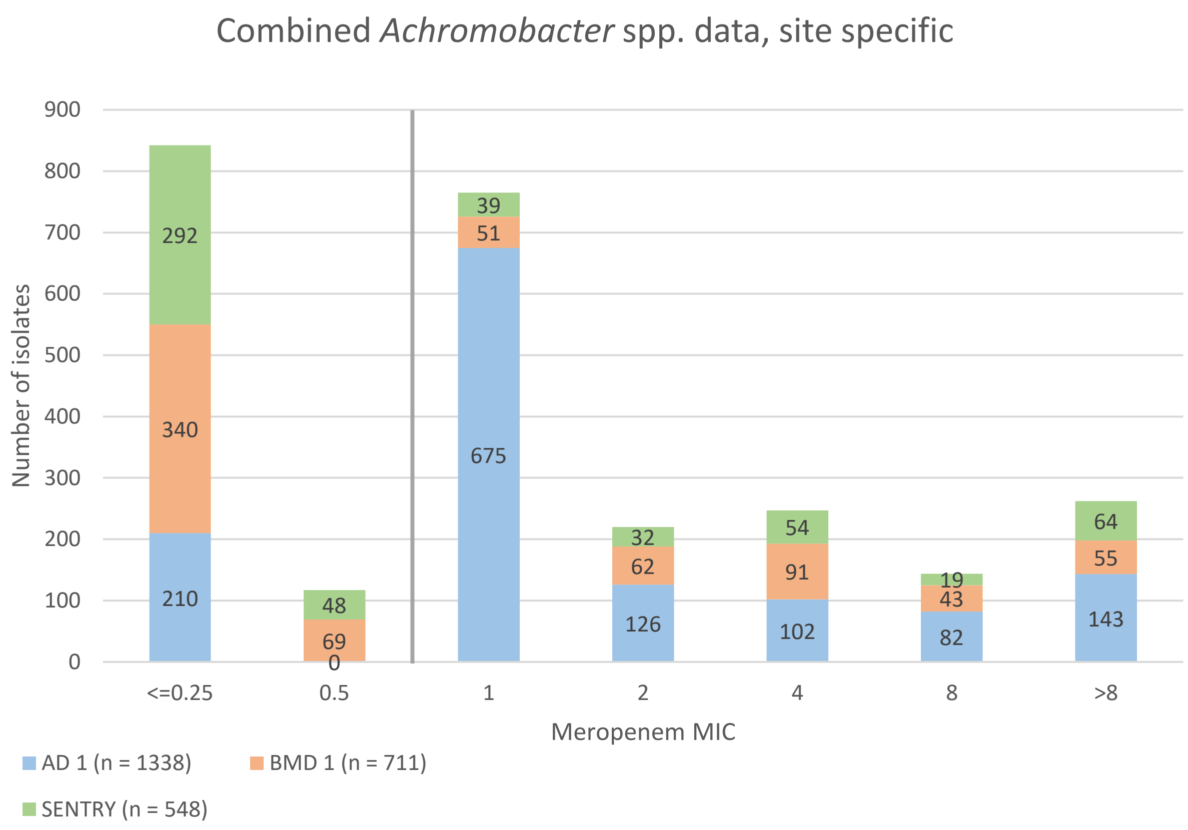

ECOFF Finder @ 97.5%: 0.5 ug/ml

Achromobacter spp. Meropenem MIC data

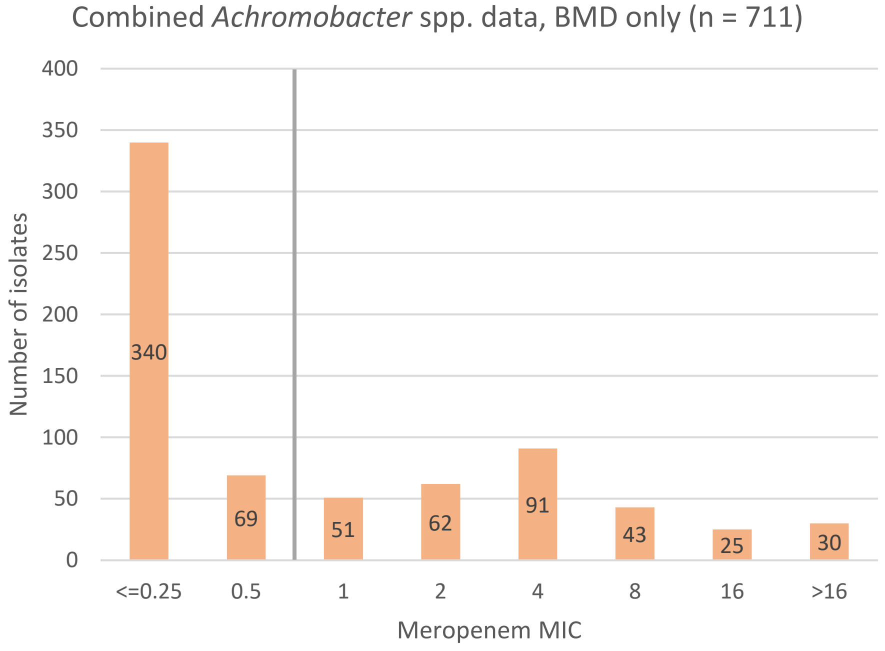

ECOFF Finder @ 97.5%: 0.5 ug/ml

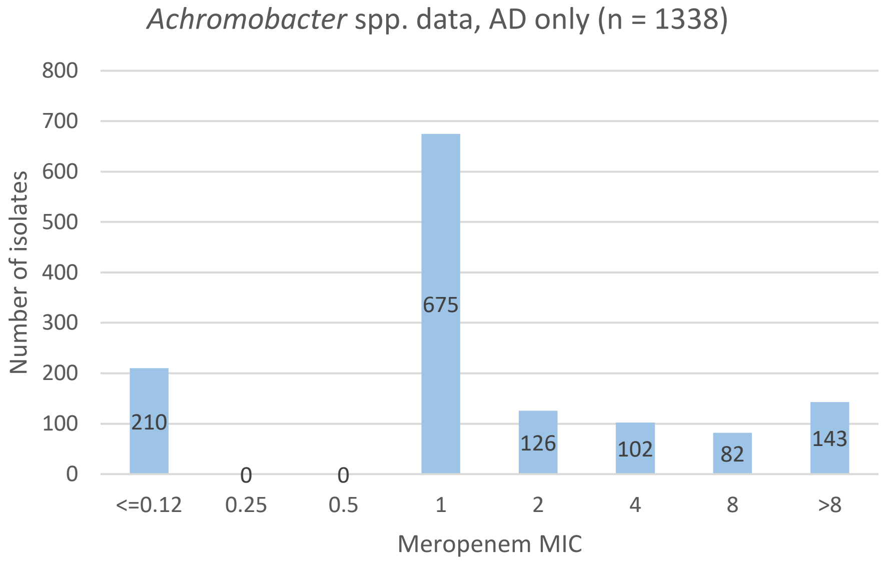

ECV could not be calculated

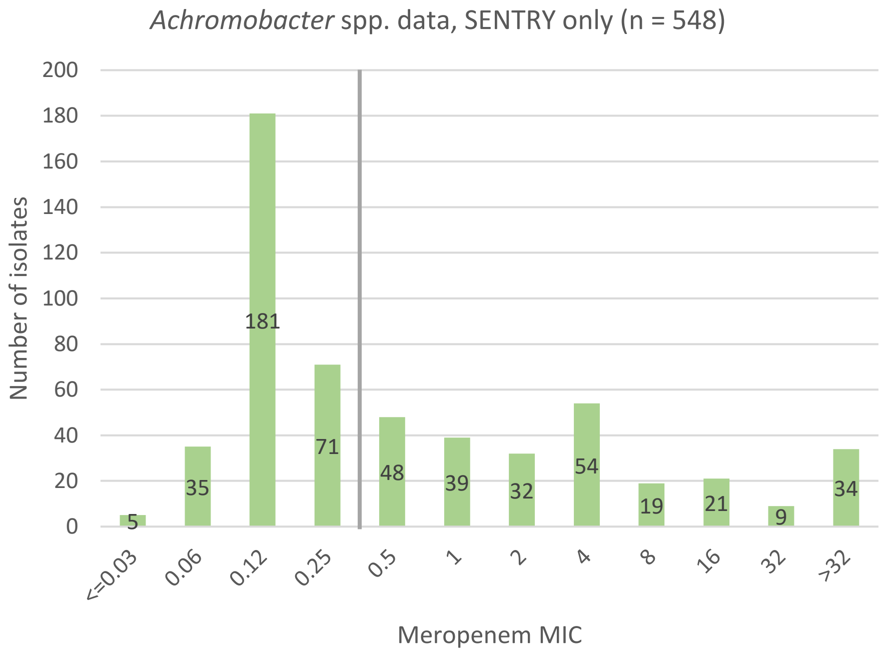

ECV could not be calculated

Achromobacter spp. Piperacillin-tazobactam MIC data (n = 2755)

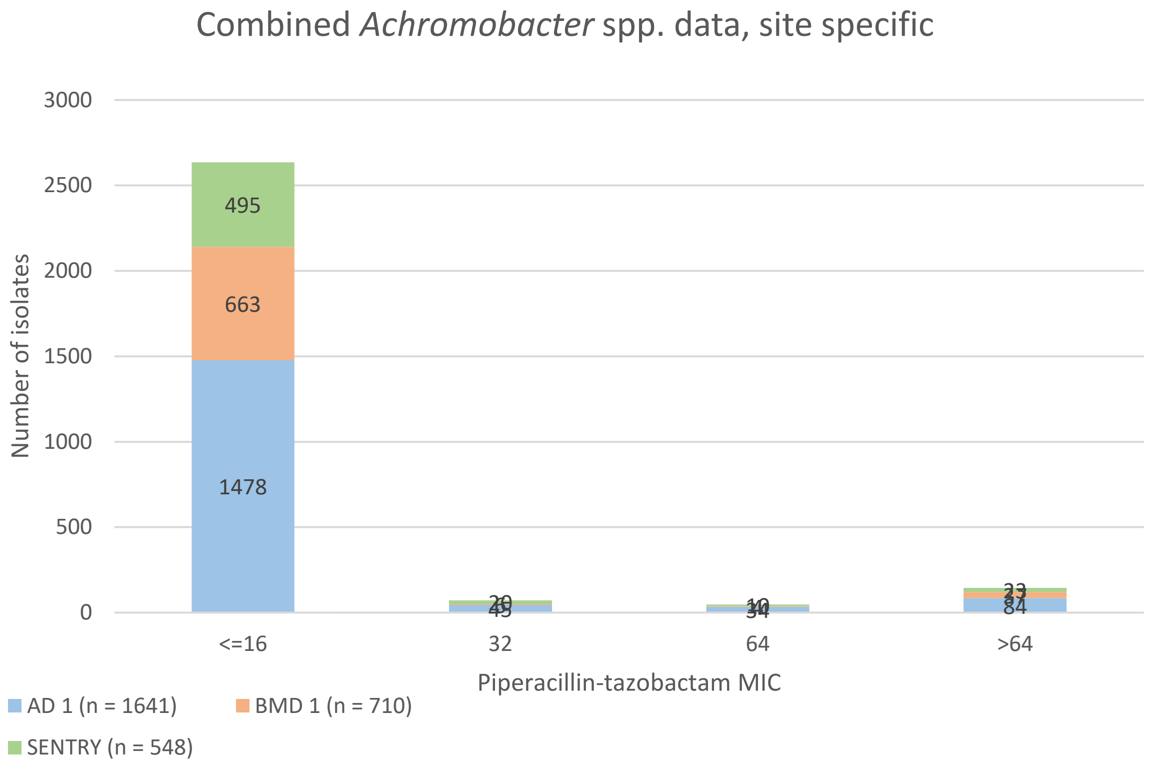

ECV could not be calculated

Achromobacter spp. Piperacillin-tazobactam MIC data

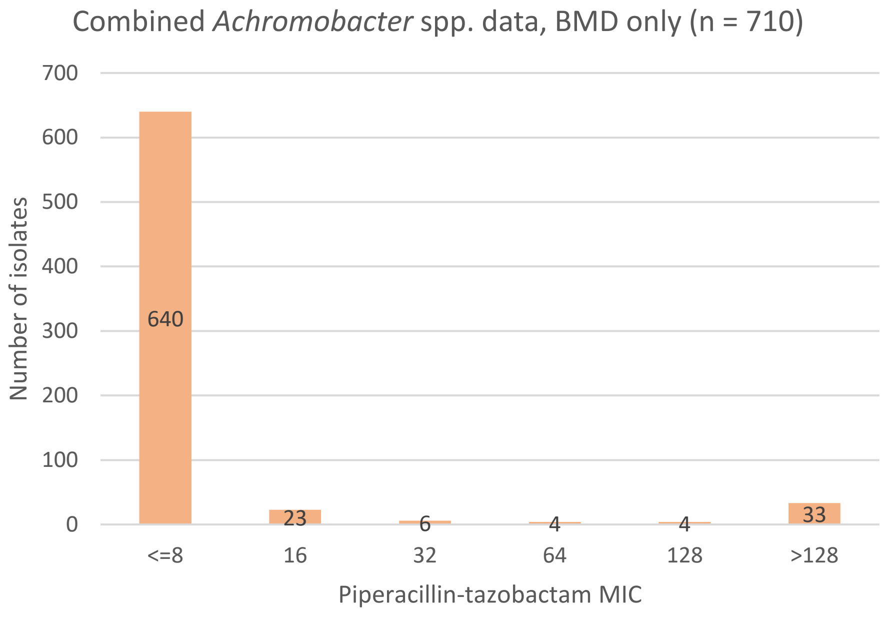

ECV could not be calculated

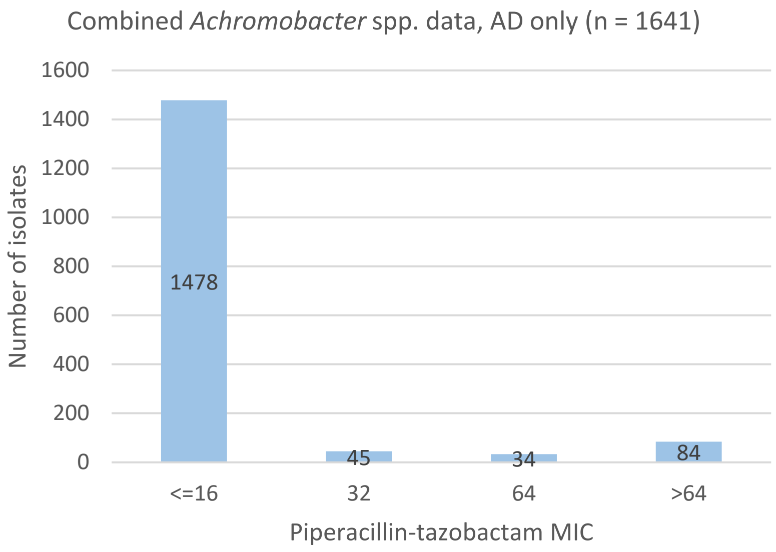

ECV could not be calculated

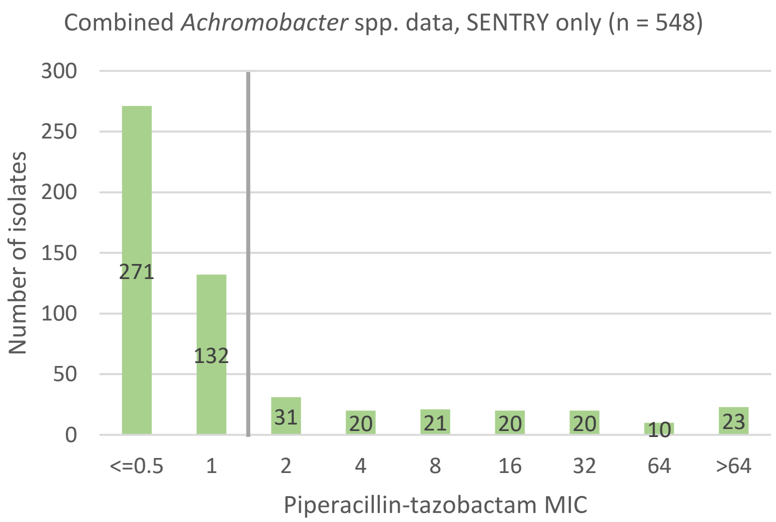

ECOFF Finder @ 97.5%: 1 ug/ml

Achromobacter spp. Trimethoprim-sulfamethoxazole MIC data (n = 2896)

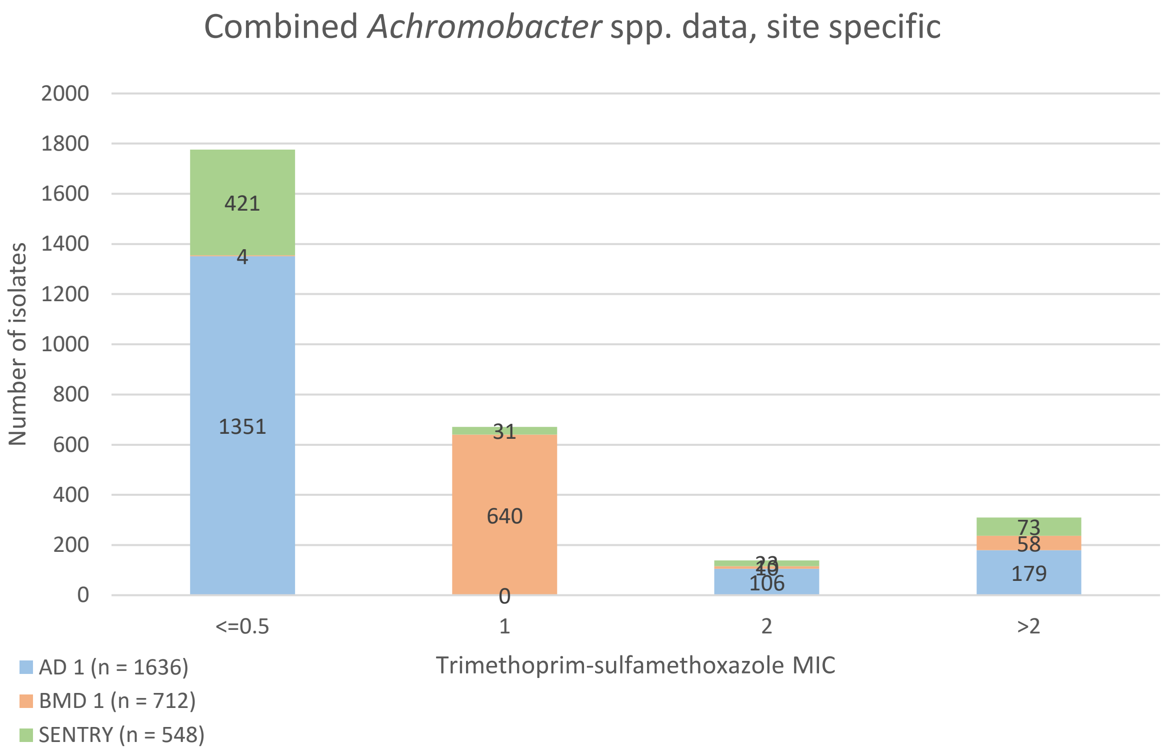

Achromobacter spp. Trimethoprim-sulfamethoxazole MIC data

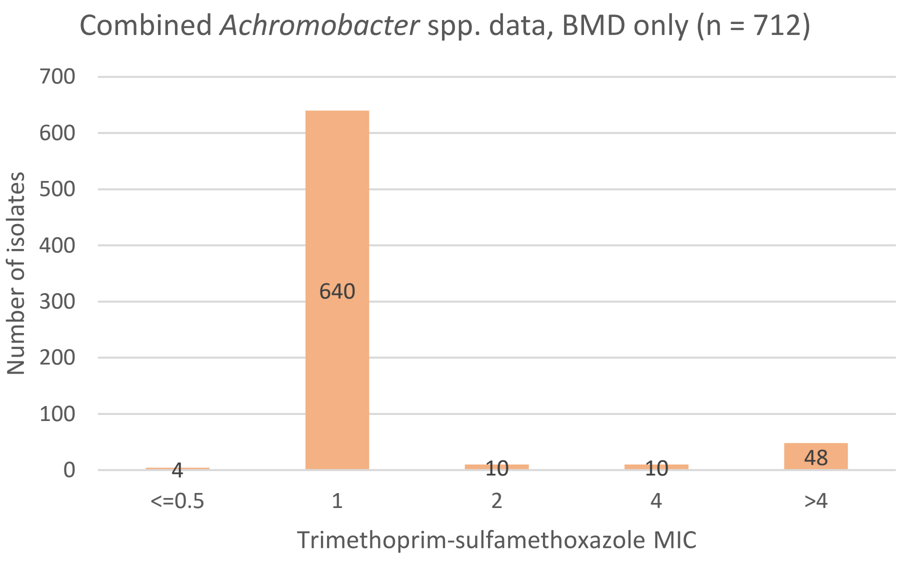

ECV could not be calculated

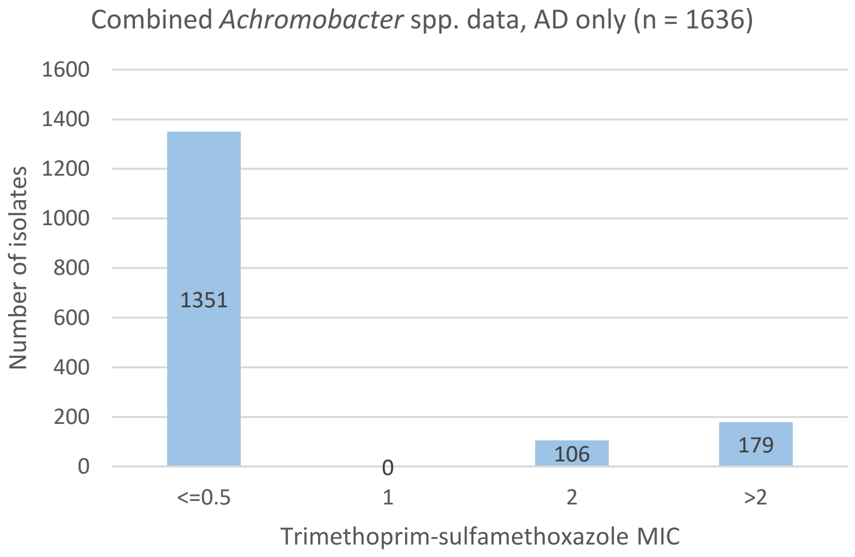

ECV could not be calculated

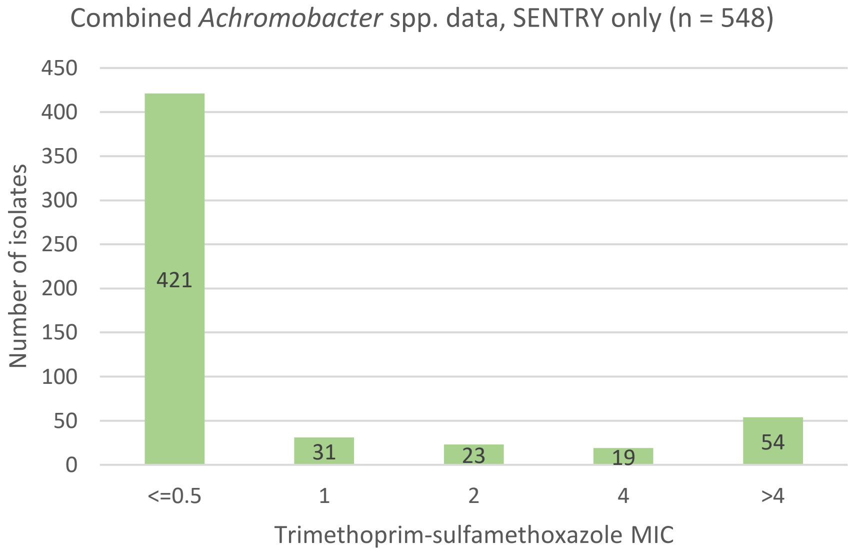

ECV could not be calculated
